# Supplementary material for: Causal Relationship between Aging and Anorexia Nervosa: A White-Matter-Microstructure-Mediated Mendelian Randomization Analysis
Source: Biomedicines. 2024 Aug 16;12(8):1874. doi: 10.3390/biomedicines12081874 (PMC11351342; doi:10.3390/biomedicines12081874)
Supplement: Supplementary file 1 [file biomedicines-12-01874-s001.zip › Supplementary Figure/Figure S7/Single SNP plot of MR analysis for ubm-b-1904 to AN.pdf]

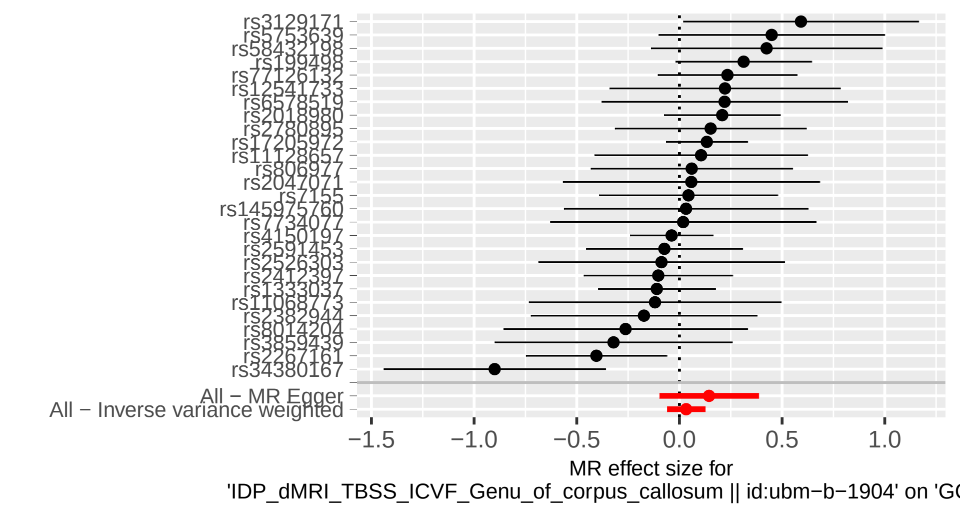

rs3129171
rs5753639
rs58432198
rs199498
rs77126132
rs12541733
rs6578519
rs2018980
rs2780895
rs17205972
rs11128657
rs806977
rs2047071
rs7155
rs145975760
rs7734077
rs4150197
rs2591453
rs2526303
rs2412397
rs1333037
rs11068773
rs2382944
rs8014204
rs3859439
rs2267161
rs34380167
All − MR Egger
All − Inverse variance weighted
−1.5
−1.0
−0.5
0.0
0.5
1.0
MR effect size for
'IDP_dMRI_TBSS_ICVF_Genu_of_corpus_callosum || id:ubm−b−1904' on 'G
